# Prognostic and clinical heterogeneity of PD1 and PD-L1- immunohistochemical scores in endometrial cancers

**DOI:** 10.1007/s00404-024-07862-y

**Published:** 2025-01-24

**Authors:** L. Proppe, T. Jagomast, S. Beume, F. Köster, K. Bräutigam, A. Rody, S. Perner, F. Hemptenmacher, J. Ribbat-Idel, L. C. Hanker

**Affiliations:** 1 Department of Gynecology and Obstetrics, University Medical Center Schleswig-Holstein, Campus-Lübeck, Lübeck, Germany; 2 Institute of Pathology, University of Luebeck, Luebeck, Germany; 3 Department of Gynecology and Gynecologic Oncology, University Medical Center Hamburg-Eppendorf, Martinistraße 52, 20246 Hamburg, Germany; 4 Department of Gynecology and Obstetrics, University Medical Center, Muenster, Germany

**Keywords:** Endometrial cancer, Molecular subgroups, Immunohistochemistry, PD1-inhibition

## Abstract

**Introduction:**

PD1/PD-L1 inhibition (ICi) has recently become a new standard of care for patients with advanced MMR-deficient (MMRd) endometrial cancers. Nevertheless, response to immunotherapy is more complex than the presence of a single biomarker and therefore it remains challenging to predict patients response to ICi beyond MMRd tumors. Elevated PD-L1 expression (CPS ≥ 1) is often used as a prognostic marker as well as a predictive biomarker of response to ICi in different tumor types. In a retrospective, patient derived study, we analyzed PD1- and PD-L1 staining and correlated the results of different scores to clinical data to evaluate the prognostic impact of these scores.

**Materials and methods:**

Immunohistochemical analysis of the receptor PD1 and the receptor ligand PD-L1 were performed on TMAs of primary paraffin‑embedded tumor samples. All patients were treated for primary endometrial cancer in the Department of Gynecology and Obstetrics, University Medical Center Schleswig–Holstein, Campus-Lübeck, Germany between the years 2006–2018. The evaluation and determination of the tumor proportion scoring (TPS), the combined positive score (CPS) and the immune cell scoring (IC) was automatically assessed semi-quantitatively, and results were correlated with clinicopathological characteristics and survival.

**Results:**

130 samples were evaluable and 64% showed a positivity (IC > 0) for the receptor PD1 and 56% for the receptor ligand PD-L1. Patients with a PD1 IC Score ≥ 1 showed a significant longer disease-free survival of 140 months (95% confidence interval (CI): 124–158) compared to patients with a lower IC < 1 for PD1 of 89 months (95% confidence interval (CI): 69–110); *p* = 0.017). Furthermore, the disease-free survival for patients with a CPS ≥ 5 for PD1 was longer (153.7 months (95% confidence interval (CI): 134–173.6) vs. 98.6 months (95% confidence interval (CI): 83–114); *p* = 0.036). Additionally, a PD1 CPS ≥ 5 showed a better overall survival but the result was not statistically significant. No difference in survival was found between patients with PD-L1 higher or lower than CPS 5.

**Conclusion:**

In this study we pointed out that there are significant clinical differences among several immunohistochemical scoring systems. In our trial, a PD1-positivity with CPS ≥ 5 and IC ≥ 1 were significantly associated to a better disease-free survival while there was no association with TPS. The PD1-IC scoring was associated with MMRd while the TPS scoring was not. Therefore, PD1-IC could be more appropriate for endometrial carcinomas compared to TPS and could also add prognostic information beside the more established PD-L1-staining. Further prospective studies are needed for a validation of these scores in combination with other biomarkers.

**Supplementary Information:**

The online version contains supplementary material available at 10.1007/s00404-024-07862-y.

## What does this study add to the clinical work

**Table:** 

This analysis demonstrates that different PD1 and PD-L1 scoring systems show different correlations with clinical data. This highlights the strong relation of pathology and gynecologic oncology and suggests that PD1-IC might be a particularly appropriate immunohistochemical marker in endometrial cancer.	

## Introduction

Endometrial cancer is the most common gynecological cancer in developed countries with increasing incidences and mortality rates over the past decades. Although, survival rates are excellent after early stage detection of the disease there are significant amounts of deaths in the recurrent and advanced disease setting, resulting in poor prognosis [1–3]. Recently, the establishment of the molecular classification of endometrial cancers led to four distinct molecular subgroups introducing new therapeutic options. The group of MMR-deficient (MMRd) endometrial cancers shows, compared to the no special molecular profile type (NSMP) and TP53 abnormal (p53abn) subgroup, a high tumor mutational burden (TMB), rendering these tumors highly responsive to immune checkpoint therapy [4]. The subgroup POLE (polymerase-e mutated) presents with an even higher TMB and the best prognosis among all four subgroups, suggesting an unnecessity of adjuvant treatment at all. MMRd predicts a relatively poor survival in endometrioid endometrial cancer compared to NSMP tumors with estrogen receptor (ER) expression and the group of POLE tumors [4, 5]. Approximately 20–40% of all endometrial cancers present a MMRd which can be detected by immunohistochemistry (IHC) after staining MLH1, PMS2, MSH2 or MSH6 or by analyzing the frequency of microsatellite instabilities (MSI) of genomic DNA (high vs low) [2, 6].

The PD1/PD-L1 signaling pathway plays a crucial role in carcinogenesis because it facilitates tumor evasion of T-cell immune surveillance by activating programmed death 1 (PD-1) signaling through upregulation of programmed death ligand 1 (PD-L1) expression on tumor cells [7]. ICi targeting PD1/PD-L1 can restore the immunogenicity of these tumors and has been demonstrated to be highly effective and well-tolerated therapy.

PD1 inhibition is one of diverse options for immune checkpoint therapy and the PD1-inhibitors Dostarlimab as well as Pembrolizumab have recently become a new standard of care for patients with an advanced MMRd endometrial cancer [8–10].

Nevertheless, it is still not possible to predict the individual therapy response before applying an immune checkpoint inhibition, especially for non-MMRd tumors. Expression of the receptor ligand PD-L1 is often used as a predictive biomarker of response to ICi in different tumor types, whereas the significance of expression of the receptor PD1 is less certain. In endometrial cancer there have been inconsistent findings concerning the prognostic and predictive effect of the expression of the receptor PD1 and the receptor ligand PD-L1 [11–13]. In terms of prediction Oaknin et al. could point out that expression of PD-L1 alone is not reliably predictive but could contribute together with TMB to better indicate responsiveness of ICi treatment in patients with advanced pretreated endometrial cancer [14].

Moreover, there exist different scores as well as laboratory techniques to evaluate PD1 and PD-L1 positivity in endometrial cancer tissue. Three major scoring systems are used for PD1 and PD-L1 IHC: the tumor proportion score (TPS), the combined positive score (CPS), and the immune cell score (IC). The CPS counts the number of PD-L1 stained tumor cells, lymphocytes, and macrophages divided by the sum of viable tumor cells, multiplied by 100. The TPS is the ratio of stained tumor cells and viable tumor cells, multiplied by 100. The IC indicates the quantity of PDL1 positive immune cells divided by the tumor area and multiplied by 100. So far, it remains unclear which scoring system reflects the reality in terms of treatment response and prognosis at its best. Evaluation on PD1 and PD-L1-positivity should be performed by an experienced pathologist as it requires routine and knowledge [15]. Although there are conflicting results on the prognostic effects of PD1/PDL-1 expression it would be crucial for patients and clinicians to know the impact of PD1 and PD-L1 on the disease-free and overall survival, especially before introducing PD1-inhibition to the adjuvant treatment of high-risk endometrial cancers.

Establishing additional predictive biomarker is an highly unmet need, especially for extending the use of immune checkpoint inhibition to adjuvant settings in early endometrial cancers or for developing more aggressive (combination) therapies that carefully balance the potential risks and benefits [16]. New molecular targets are being researched and frequently demonstrate significant impact on the survival of endometrial cancer patients [17, 18]. In this context the DUO-E trial could demonstrate that the combination of the anti-PD-L1 antibody Durvalumab to standard first-line chemotherapy, followed by Durvalumab and the PARP inhibitor Olaparib, improved outcomes in newly diagnosed advanced or recurrent endometrial cancer especially in the PD-L1–positive subgroup (TAP > 1%) [19]. In this trial another score, the tumor positivity area score (TAP) a novel visual estimation method for combined tumor cell and immune cell scoring was used [20]. In contrast to this Ramirez et al. recently revealed an advantage of ICi treatment with Pembrolizumab independently of PD-L1 expression according to CPS score in the first-line setting [21].There are limited data regarding clinicopathologic characteristics and PD1/PD-L1 status of endometrial cancer patients in respect to different scoring techniques. This study aims to analyze patient parameters as well as their association with survival data and comparing them to the scores IC, TPS and CPS of PD1/PD-L1 expression.

## Materials and methods

**Fig. 1:**
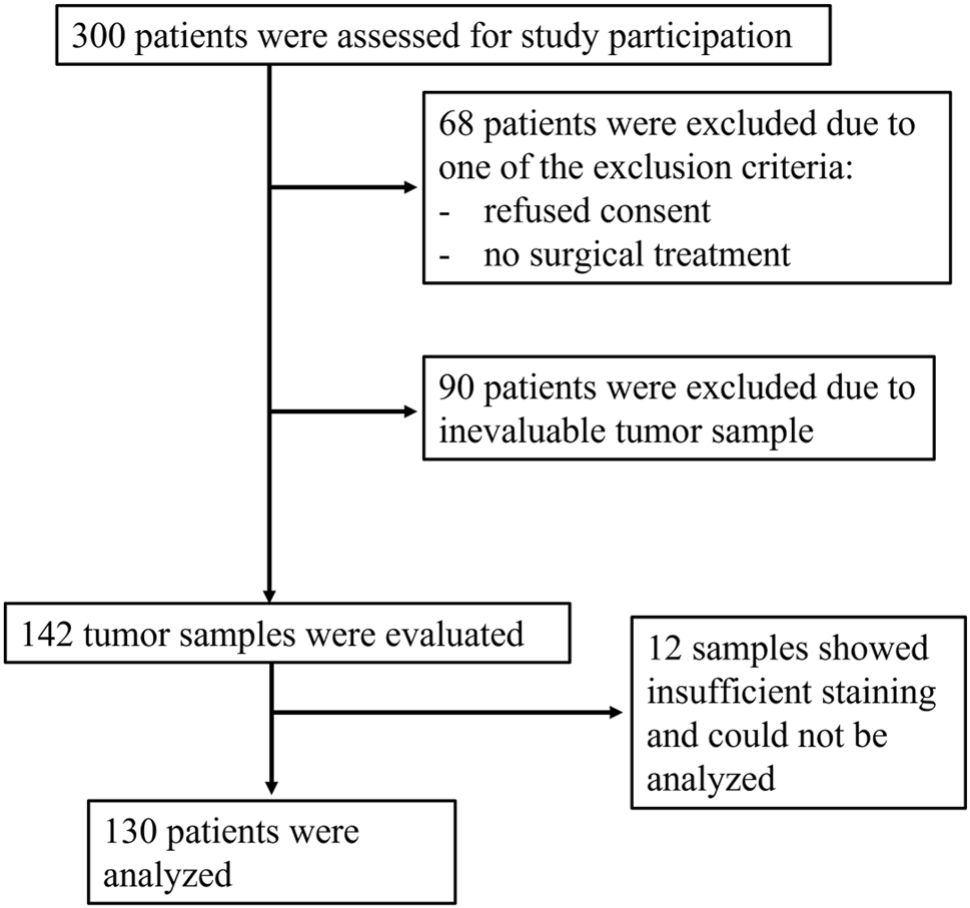
Consort diagram

Patients treated surgically for primary endometrial cancer in the Department of Gynecology and Obstetrics, University Medical Center Schleswig–Holstein, Campus-Lübeck between 2006 and 2018 were included retrospectively. The study was performed in compliance with the Helsinki Declaration and approved by the ethics committee of the University of Lübeck (19-082A). Exclusion criteria were no surgical treatment, the absence of patient consent, and the absence of adequate tumor tissue. 130 patients finally met the inclusion criteria (Fig. 1).

Medical history, details of surgery, histology, tumor stage, and postoperative data were reviewed in the patient reports. A questionnaire was used to collect data concerning the disease-free survival. These clinical data were correlated with IHC results.

To evaluate appropriate tumor content and review primary diagnosis, archived H&E slides of hysterectomy-preserved specimens were assessed by a board-certified pathologist. Tumor cell ROIs were annotated, and a core per specimen was arranged to tissue microarrays (TMAs). Each TMA consisted of up to 60 1 mm^2^ samples, thereof up to 6 non-neoplastic endometrium and up to 54 tumor tissue samples.

The PD1 (NAT105, mouse monoclonal antibody, Cell Marque, Merck, Darmstadt) and PD-L1 (Clone 22C3, mouse monoclonal antibody, Dako, Hamburg) antibodies were used to evaluate the PD1 and PD-L1 status of each tumor sample. For each staining the IView DAB Detection Kit was used on a Ventana Bench Mark automated staining system (Roche, Basel, Switzerland).

The stained TMAs were then scanned using the Ventana iScan HT scanner (Ventana Tucson, AZ, USA). The samples were computerized and semi-automatically analyzed using the Definiens Tissue Studio software (Definiens Developer XD 2.0, Definiens AG, Munich, Germany). PD1 and PD-L1 expression was analyzed via different immunohistochemical scoring systems which were TPS (tumor proportion scoring) ≥ 1, IC (immune cell scoring) ≥ 1 and CPS (combined positive score) ≥ 5, respectively. The remaining immunohistochemical analyses were performed according to standard procedures in the Department of Pathology at University Hospital Luebeck. The Ki-67 staining was evaluated using Definiens Software, while p53 and MMR staining was analyzed using the eyeballing system. Every step was supervised by an experienced pathologist, and clinical routine methods were applied.

Statistical analyses were performed using IBM SPSS Statistics for Windows (SPSS Statistics, v 27, IBM Corp., Armonk, NY, USA). The immunohistochemical staining intensity has been correlated to clinicopathological characteristics and survival data. We hypothesized that some scoring systems may be more appropriate with regard to the survival times in patients with endometrial cancer than others. We used the *Chi*^*2*^*-test* to compare frequencies among groups. To analyze survival data, Kaplan–Meier curves and the log-rank test were applied. For multivariate analyses the Cox regression method was used. A *p*-value below or equal to 0.05 was considered significant.

## Results

We evaluated the impact of PD1 receptor and PD-L1 receptor ligand status according to the three scoring systems CPS, TPS, IC on different clinical characteristics and on survival data. Mean age of all patients was 66.9 ± 12.3 years, the mean BMI amounted to 30.3 ± 8.6 kg/m^2^ (SD). The majority of the patients was classified in tumor stages FIGO (2009) I or II. 62.7% of the patients received a systematic lymphadenectomy, but only 15 patients (11.5%) finally had lymph node metastases. According to the pathologic reports, 84.5% of the tumors had an endometrioid histology and 60.6% of the patients were surgically treated by laparoscopy.

Baseline clinical characteristics of all patients are presented in Table 1.

**Table 1:** Baseline characteristics and clinical data of patients (n = 130)

Characteristic	Classification	No. of patients (%)
Age (years)	> 60	93 (71.5%)
	≤ 60	37 (28.5%)
FIGO	I-II	97 (74. 6%)
	III-IV	31 (23.8%)
	Unknown	2 (1.6%)
BMI (kg/m^2^)	> 30	50 (38.5%)
	≤ 30	70 (53.8%)
	Unknown	10 (7.7%)
Surgery technique	Laparoscopy/vaginal approach	77 (59.2%)
	Laparotomy	48 (36.9%)
	Unknown	3 (3.9%)
Lymph node metastases	Yes	15 (11.5%)
	No	68 (52.3%)
	Unknown	47 (36.2%)
Grading	Low grade (G1)	66 (50.8%)
	High grade (G2 or 3)	61 (46.9%)
	Unknown	3 (2.3%)
p53 status	p53 aberrant	55 (42.3%)
	p53 wild type	73 (56.2%)
	Unknown	2 (1.5%)
MMR status	MMR deficient	73 (56.2%)
	MMR proficient	55 (42.3%)
	Unknown	2 (1.5%)
Ki -67 status	Ki-67 ≥ 25%	30 (23.1%)
	Ki-67 < 25%	89 (68.5%)
	Unknown	11 (8.4%)
Histopathology	Endometrioid carcinoma	108 (83.1%)
	Others (e.g. serous or clear cell carcinomas)	21 (16.2%)
	Unknown	1 (0.7%)

### PD1 expression

130 tumor samples underwent evaluation following PD1 staining. Of these, 78 samples tested PD1 positive based on the IC scoring (IC ≥ 1). Using CPS, 31 samples were considered positive. For the TPS, 5 samples revealed a positive PD1 status. Upon assessing the IC score, no significant associations were identified between histopathological or clinical parameters and the PD1 staining (Table 2). The clinical parameters are analyzed in Table 2 for PD1-positivity according to the IC score.

**Table 2:** Data of patients dependent on the PD1 IC score (n = 130)

Characteristic	Classification	Amount of patients with PD1-IC ≥ 1 (*n* = 78 (60%))	Amount of patients with PD1-IC < 1 (*n* = 52 (40%))
Age (years)	> 60	56 (43.1%)	37 (28.5%)
	≤ 60	22 (16.9%)	15 (11.5%)
FIGO	I–II	57 (43.8%)	41 (31.5%)
	III–IV	20 (15.4%)	10 (7.7%)
	Unknown	2 (1.6%)	
MMR status	MMR deficient	47 (36.2%)	27 (20.8%)
	MMR proficient	30 23.1%)	25 (19.2%)
	Unknown	2 (1.5%)	
p53 status	p53 aberrant	32 (24.6%)	23 (17.7%)
	p53 wild type	45 (34.6%)	29 (22.3%)
	Unknown	2 (1.5%)	
Ki -67 status	Ki-67 ≥ 25%	18 (13.8%)	12 (9.2%)
	Ki-67 < 25%	52 (40%)	37 (28.5%
	Unknown	11 (8.4%)	
Grading	Low grade	36 (27.7%)	29 (22.3%)
	High grade	41 (31.5%)	21 (16.2%)
	Unknown	3 (2.3%)	
Histopathology	Endometrioid carcinoma	65 (50%)	43 (33.1%)
	Others (e.g. serous or clear cell carcinomas)	12 (9.2%)	9 (9.9%)
	Unknown	1 (0.7%)	

No significant differences between PD1-IC level and patient characteristics were found

The statistical analyses revealed that there are only marginal differences between the three scores concerning the different clinical and histopathologic parameters for PD1 (Table s1, supplemental information).

### PD-L1 expression

**Fig. 2:**
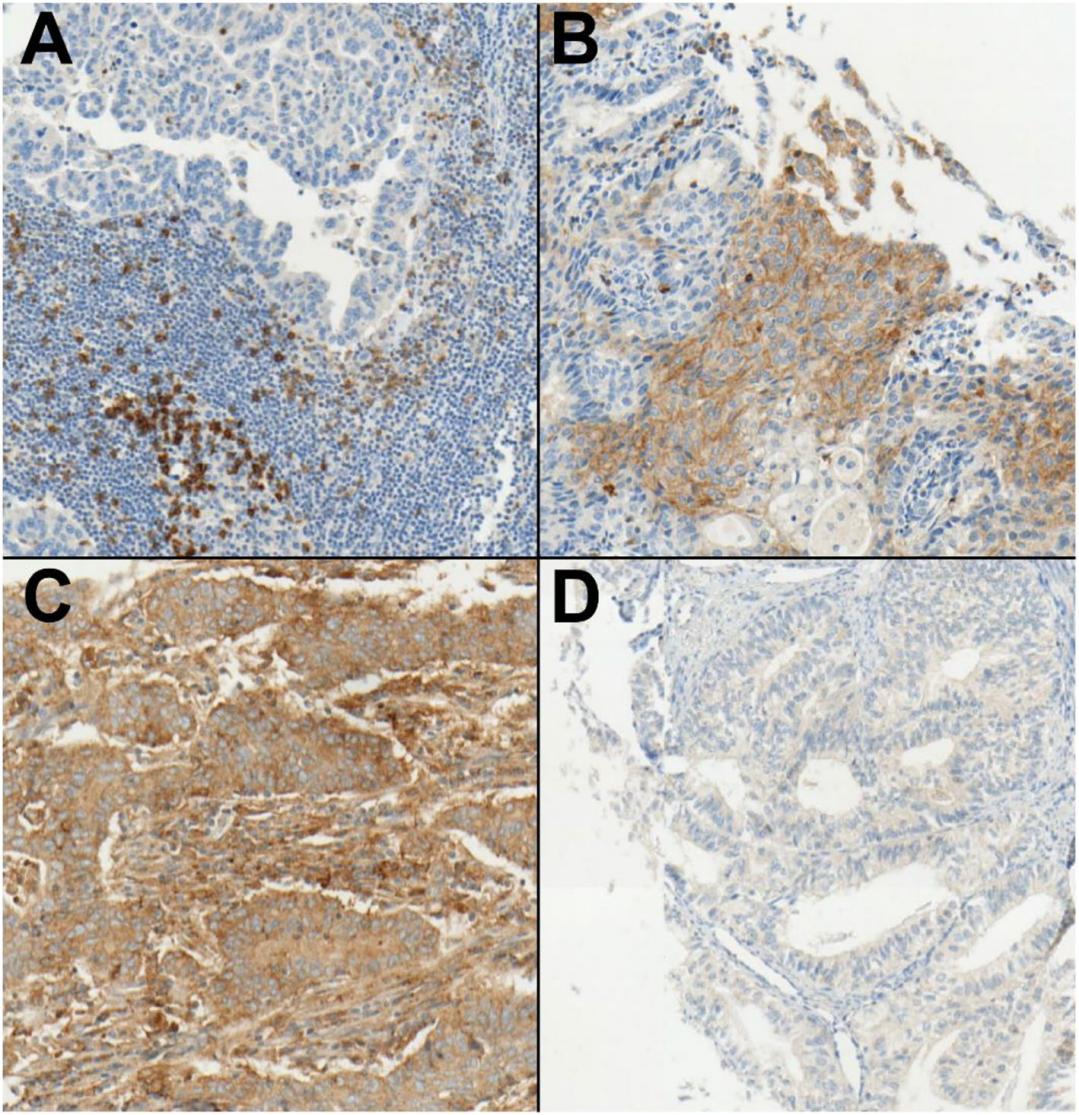
Examples of IHC in FFPE tissues from endometrial carcinoma patients with PD1 (**A**, **B**) and PD-L1 (**C**, **D**) detection. **A** This core shows positive staining of immune cells for PD1 antibody while tumor cells are negative. **B** This core shows positive staining of immune cells for PD1 antibody and an area of PD1 positive tumor cells. **C** This core shows intense staining of tumor cells for PD-L1. **D** This core shows negative staining for PD-L1

127 tumor samples underwent evaluation following PD-L1 staining. Of these, 63 samples tested PD-L1 positive based on the IC scoring (IC ≥ 1). Using CPS, 27 samples were considered positive. For the TPS, 25 samples revealed a positive PD-L1 status. Statistical analyses revealed dependencies of the PD-L1 status and the categories of age, grading and histopathology. These results were not consistent when using the different scores IC, TPS or CPS, as depicted in Table 3. Further survival results for PD-L1-positivity are listed in the supplemental information. We refrained from calculating statistical significances due to small group sizes. However, it is notable that more patients over 60 years old were PD-L1 positive compared to those 60 years old or younger. Moreover, in terms of grading categories, the majority of patients with a PD-L1 positive tumor were diagnosed with a high-grade tumor (Fig. 2).

**Table 3:** Data of patients dependent on a positive PD-L1 status (n = 127)

Characteristic	Category	Amount of patients with PD-L1-IC ≥ 1 (*n* = 63, (49.6%))	Amount of patients with PD-L1-CPS ≥ 5 (*n* = 27, (21.3%))	Amount of patients with PD-L1-TPS ≥ 1 (*n* = 25, (19.7%))
Age (years)	> 60	50 (39.4%)	23 (18.1%)	20 (15.7%)
	≤ 60	12 (9.4%)	4 (3.1%)	5 (3.9%)
FIGO	I–II	46 (36.2%)	17 (13.4%)	15 (11.8%)
	III–IV	15 (11.8%)	9 (7.1%)	9 (7.1%)
MMR status	MMR deficient	37 (29.1%)	13 (10.2%)	14 (11%)
	MMR proficient	25 (19.7%)	14 (11%)	11 (8.7%)
p53 status	p53 aberrant	24 (18.9%)	14 (11%)	13 (10.2%)
	p53 wild type	38 (29.9%)	12 (9.4%)	11 (8.7%)
Ki -67 status	Ki-67 ≥ 25%	13 (10.2%)	4 (3.1%)	7 (5.5%)
	Ki-67 < 25%	45 (35.4%)	21 (16.5%)	15 (11.8%)
Grading	G1	30 (23.6%)	8 (6.3%)	7 (5.5%)
	G2 or G3	31 (23.6%)	18 (14.2%)	16 (12.6%)
Histopatho-logy	Endometrioid carcinoma	50 (39.4%)	17 (13.4)	15 (11.8%)
	Others (e.g. serous or clear cell carcinomas)	13 (10.2%)	10 (7.9%)	10 (7.9%)

### PD1 survival analysis

**Fig. 3:**
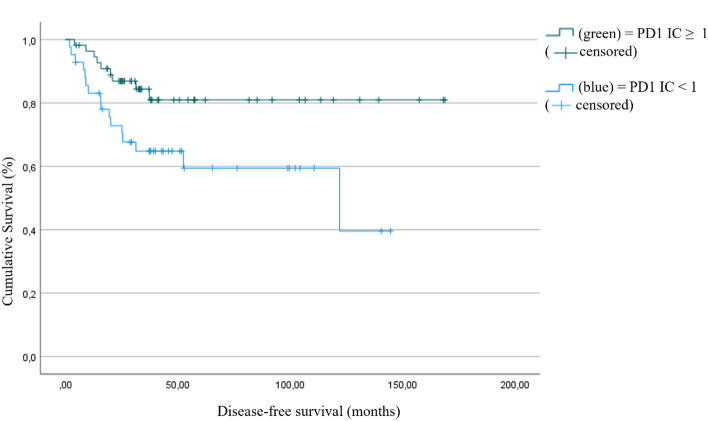
Disease-free survival as a function of PD1 positivity, defined as IC ≥ 1 (*p* = 0.017)

The disease-free survival is significantly longer in patients with PD1 expressing (IC score ≥ 1) immune cells (140.6 months; 95% CI: 123.5–157.6) than in those with low PD1 (89.4 months; 95% CI: 68.8–109.9) immune cell expression. The estimated disease-free survival differed by 51.2 months (*p* = 0.017; Fig. 3).

**Fig. 4:**
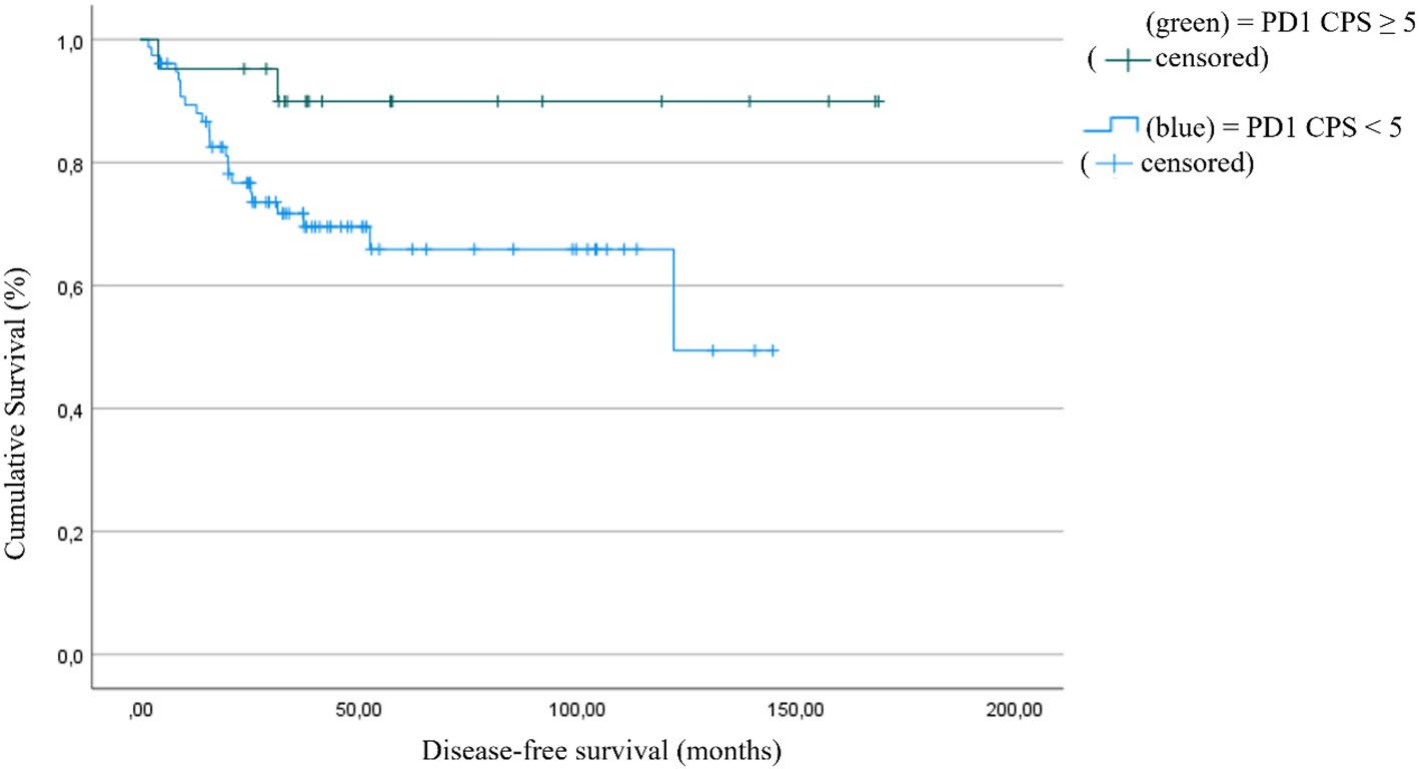
Disease-free survival as a function of PD1 positivity, defined as CPS ≥ 5 (*p* = (153.7 months (95% CI:133.8–173.6) vs. 98.6 months (95% CI: 82.9–114.3), (*p* = 0.036)

We could point out consistent results in regard to the CPS Score. The disease-free survival is significantly longer in patients with PD1 expression (CPS score ≥ 5) than in those without PD1 expression. The estimated disease-free survival differed by 55.1 months (153.7 months (95% CI:133.8–173.6) vs. 98.6 months (95% CI: 82.9–114.3), (*p* = 0.036; Fig. 4).

The DFS analysis for TPS did not reveal any differences according to PD1 expression.

Concerning OS analysis a PD1 CPS ≥ 5 showed a trend for a better overall survival although not statistically significant (Fig S4). According to IC or TPS scores for PD1 no significant differences for OS were found.

### PD-L1 survival analysis

For patients with the receptor ligand PD-L1 IC score ≥ 1 the disease-free and overall survival showed a trend for a better survival. Applying the TPS score, there was no difference observed in either OS or DFS. A trend towards better overall survival in PD-L1 positive cases, defined as CPS ≥ 5, has been observed while no difference was noted in terms of disease-free survival (see supplemental information).

### PD1 expression according to molecular subgroups MMRd and p53abn

The molecular subgroup MMRd is therapeutically of great interest and indicates tumor sensitivity to PD1-inhibition. In this cohort, MMRd tumors are significantly more often PD1 positive than PD1 negative ((IC ≥ 1 or CPS ≥ 5, *p* = 0.016 and *p* = 0.026, respectively). When analyzing the p53abn subgroup, there was no significant association for PD1-positivity or PD-L1-positivity. Table 3 presents a crosstabulation based on tumors considered PD1-positive, with an IC-score ≥ 1, as this appears to influence disease-free survival, as shown in Fig. 3 (Table 4).

**Table 4:** p53-status is not significantly associated with PD1-positivity (IC-score)

PD1-status (IC-score)	p53 wild type tumor	p53 aberrant tumor	Total
Negative	29	23	52
Positive	45	32	77
Total	74	55	129

## Discussion

In the above mentioned retrospective analysis, we demonstrated that PD1-expression in endometrial cancer cells could influence disease-free survival. Moreover, our examination suggests that the IC score and CPS may be more suitable indicators than the Tumor Proportion Score (TPS). Similar to our results Kim et al. were also able to reveal an association of PDL-1/PD1 expression with survival data.Their study demonstrates that patients with relatively high PD-1 expression had a more favorable overall survival compared to those classified as low PD-1 expressers. However, it is worth noting that in the analysis by Kim et al., a high PD-L1 expression (IC score) was associated with an unfavorable progression-free survival, introducing some controversy in their results [22].

The analysis of PD-1 and PD-L1 expression in endometrial cancer tissue and its potential prognostic significance remains unclear in the literature. The role of the PD1/PD-L1 pathway in the carcinogenesis and its role in the immune escape mechanisms of endometrial cancer are not fully understood. Moreover, Siraj et al. found that PD-L1 expression in endometrial cancer is associated to lymph nodes metastases, suggesting that PD-L1 may additionally serve as an independent predictive biomarker [23].

Many solid cancers evade the endogenous immune response by expressing aberrant PD1 receptors. If patients with such tumors do not receive immune checkpoint inhibition therapy, the tumors may progress differently compared to those with wild type PD1 expression.

Our study is warranted to explore the implications of PD1- and PD-L1 expression, shedding light on their possible prognostic value, which could have crucial implications for the management and treatment of endometrial cancer patients. If we could predict more precisely treatment response and prognosis, we would be able to de-escalate therapies and spare patients from potential adverse events. Additionally, the various existing PD1 and PD-L1 scoring systems suggest that the clinical implications of some studies might partly depend on which scoring system is used. Currently, the poor validation of PD1 or PD-L1 expression scores is another component which complicates essentially treatment response prediction and cross-study comparisons.When analyzing endometrial cancer subgroups according to current guidelines, several associations can be observed. Jin et al. demonstrated that 15% of endometrial clear cell carcinomas, which partially overlap with p53 aberrant carcinomas, are categorized as “PD-L1-positive”. This becomes particularly significant due to the poorer prognosis associated with this subgroup in comparison to other endometrial cancer subgroups. Estimating therapy response and individual prognosis is of paramount importance in such cases [24]. In our present study, we did not find a significant association with p53 aberrant tumors. However, in contrast to reports in the literature, we identified a relatively high number of patients with p53 aberrant or mismatch repair deficient tumors. This variation could potentially be attributed to factors such as prolonged storage and challenges associated with immunohistochemical staining.

As expected, the MMRd tumor subgroup is more frequently associated with PD1-positive tumors (based on the IC score) when compared to non-MMRd tumors. This correlation aligns with previously published data in the literature although it is also important to note that other studies reveal controversial findings, indicating a lack of association [22, 24–28].

The exact role of PD-1 or PD-L1 expression in tumor dissemination remains a topic in need of further investigation. In our analysis, we did not identify any significant association between PD-1 or PD-L1 expression and FIGO stages. Interestingly, conflicting findings have been reported using other cohorts suggesting a link between PD-L1 or PD1 expression and metastatic spread [12, 23]. In one study, PD-L1 expression in endometrial cancer was recognized as an independent marker for lymphatic metastasis. Furthermore, they reported no significant association between PD-L1 expression and POLE mutation [23]. In our cohort, we did not analyze the POLE status of the tissue samples. Further research is needed to clarify these complex relationships.

As previously highlighted, the PD1/PD-L1 pathway plays a crucial role in the immune evasion mechanisms of cancer tissue, serving as a target for immune checkpoint inhibition. This is potentially bearing significant prognostic implications, especially in recurrent endometrial cancer with the potential of a great improvement of the survival [29]. Notably, due to its strong correlation with the T-cell immune system, PD1 expression is associated with other immune markers such as CD3, and both predict a more favorable relapse-free survival when overexpressed [30, 31]. In recent years, the critical influence of the immune system in the tumor microenvironment on both the development of cancer (carcinogenesis) and its response to treatment has been recognized. They seem just as important as the cancer cells themselves in affecting the course of the disease and therapeutic outcomes. Among these immune cells, TILs play a vital role, as they express PD1 receptors on their surface. Without the presence of TILs within the tumor, immune checkpoint inhibition therapies—such as PD1 and PD-L1 inhibitors—cannot effectively block the immune-suppressive signals that allow cancer cells to evade the immune response. Thus, the interaction between TILs and the tumor microenvironment is crucial for the success of these therapies. Therefore, a major limitation of this study is the absence of analysis related to tumor-infiltrating lymphocytes (TILs), which should ideally be evaluated in relation to prognoses and the expression of PD1 and PD-L1 [32].

Additionally, it is important to acknowledge the retrospective nature of this study and that it represents a single-center cohort.

A general limitation of immunohistochemical techniques is the requirement for individualized evaluation of the staining. As demonstrated by Chebib et al., PD-L1 staining and scoring are intricate, and there is a noticeable lack of standardization in this process [33]. Furthermore, there is even less methodology and analysis available for PD1 staining when compared to PD-L1 staining. Therefore, we posit that assessing PD1 staining may be even more challenging than PD-L1 staining. In our study, we did not identify any associations between PD1-Tumor Proportion Score (TPS) and disease-free or overall survival, but a strong correlation was evident between PD1-Combined Positive Score (CPS) and disease-free survival. These findings align with existing literature, suggesting that various immunohistochemical antibodies, scoring systems, and techniques can yield differing results [27]. These discrepancies as well as differences caused by the tumor itself may also account for variations in the literature regarding the prognostic impact of PD-L1 expression [28]. For instance, Zhang et al. examined patients with endometrial cancer and found improved overall survival in those with PD-L1-positive tumors (both TPS and IC score) [34]. However, in our study, we did not observe any association of PD-L1 expression and the prognosis. Considering the wide variation between the different scores demonstrated in this study, one may conclude that the Tumor Area Positivity score (TAP) warrants further investigation and establishment as it may offer a more valid scoring method The TAP score combines both tumor and immune cell positivity, reflecting the critical role of the tumor microenvironment in cancer progression and response to treatment. The method offers a comprehensive approach by considering the tumor cells and also the immune cells, being a crucial part in the tumor microenvironment. Furthermore, the TAP method is relied on visual recognition patterns, which allows both, an efficient and accurate quantification of PD1 and PD-L1 positivity. Therefore, the TAP method holds the potential to standardize the scoring of PD1 and PD-L1 in gynecologic oncology. By offering a consistent and reproducible scoring system, the reliability of immunotherapy-related biomarker research could be improved.

Doroshow et al. have demonstrated that the CPS appears to offer better reproducibility compared to the IC scoring system. Establishing a valid biomarker necessitates not only high specificity but also high reproducibility, which the CPS may help achieve [35]. We hypothesize that the CPS may be the most appropriate PD1 scoring method, showing a robust prognostic significance for patients with endometrial cancer.

Despite the now recognized molecular subgroups in accordance to the ESGO guidelines, predicting an individual prognosis or therapy response remains challenging. As demonstrated in this study and in the literature, PD1 expression may offer a potential biomarker that complements the existing subgroups [11]. In the future, there is a possibility to establish PD1 as an independent prognostic marker and tool for therapy surveillance using peripheral blood samples from endometrial cancer patients, as previously suggested by Gibney et al. and as has been studied in patients with melanoma [36]. Recently, the role of autoimmune tumor response is becoming increasingly important because the immune checkpoint inhibition has been widely used. If it would be possible to better predict therapy response, therapy-associated risks might be reduced.

Predictive biomarkers could guide alternative treatment strategies when a particular therapy is unlikely to be effective. In future projects may start by collecting tissue and blood samples from patients with endometrial cancer. From studies on melanoma patients, it’s known that blood cell counts have prognostic relevance [36]. All known PD1 and PD-L1 scoring systems should be applied to the tissue samples including the analysis of the TILs, while peripheral blood samples should also be analyzed [37].

## Conclusion

Our results show a high correlation of a positive PD1-IC and PD1-CPS score and disease-free survival. Furthermore, we showed that PD1-positive tumors are more often MMRd than MMRp, compared to PD1-negative tumors using the IC-score. This confirms the therapeutic impact of immune checkpoint inhibition in MMRd tumors. The PD-L1-status was not associated with the survival times, which is in line with other studies [27, 30]. The therapeutic impact of these results should be further investigated. We assume that the PD1 status in endometrial cancers may have significant prognostic impact. Furthermore, the PD1-IC score and PD1-CPS might be more appropriate for endometrial cancer diagnostics compared to the TPS score because it may consider the impact of tumor’s microenvironment. Due to significant differences between the scoring systems, it may be worthwhile to explore a combination with further biomarkers such as TMB. Furthermore, new scoring systems like TAP should undergo further analysis.

## Supplementary Information

Below is the link to the electronic supplementary material.

Supplementary file1 (DOCX 675 KB)

## Data Availability

Further data are available in the supplemental information and on request.
