# Supplementary material for: Prognostic and clinical heterogeneity of PD1 and PD-L1- immunohistochemical scores in endometrial cancers
Source: Arch Gynecol Obstet. 2025 Jan 24;311(5):1395–405. doi: 10.1007/s00404-024-07862-y (PMC12033092; doi:10.1007/s00404-024-07862-y)
Supplement: Supplementary file 1 — Supplementary file1 (DOCX 675 KB) [file 404_2024_7862_MOESM1_ESM.docx]

**Prognostic and Clinical Heterogeneity of PD1 and PD-L1- Immunohistochemical Scores in Endometrial Cancers**

Proppe L^1,4*^, Jagomast T.^2^, Beume S.^1^, Köster, F.^1^, Bräutigam, K.^1^, Rody A.^1^, Perner S.^2^, Ribbat-Idel J.^2^ Hemptenmacher F.^1^ Hanker LC^1^

^1^Department of Gynecology and Obstetrics, University Medical Center Schleswig-Holstein, Campus-Lübeck, Lübeck, Germany ^2^Department of Pathology, University Medical Center Schleswig-Holstein, Campus-Lübeck, Lübeck, Germany ^3^Department of Gynecology and Obstetrics, University Medical Center Charité Berlin, Berlin, Germany^4^Department of Gynecology and Gynecologic Oncology, University Medical Center Hamburg-Eppendorf, Germany

**Supplemental Information**

We aim to provide a comprehensive scientific work, and as such, we have decided to publish the remaining Kaplan-Meier curves pertaining to patient data with PD1-positive tumors based on the TPS score (≥ 1, Fig S1 and S2) and those demonstrating the overall survival based on the IC and CPS scores (Fig S3, S4). Moreover, the clinical data dependent on the different PD1-scores are depicted in Table 1. The statistical validity of these analyses is limited due to the small number of positive tumor samples identified to be PD1-positive (TPS ≥ 1).

Table S1. Data of patients dependent on a positive PD1 status (*n* = 114).

| Characteristic | Classification | Amount of patients with  PD1-IC ≥ 1  (*n* = 78,  (68.4 %)) | Amount of patients with  PD1-CPS ≥ 5  (*n* = 31,  (27.2 %)) | Amount of patients with  PD1-TPS ≥ 1  (*n* = 5,  (4.3 %)) |
| --- | --- | --- | --- | --- |
| Age (years) | > 60 | 56 (49.1 %) | 21 (18.4 %) | 1 (0.9 %) |
|  | ≤ 60 | 22 (19.3 %) | 10 (8.8 %) | 4 (3.5 %) |
| FIGO | I-II | 57 (50 %) | 20 (17.5 %) | 4 (3.5 %) |
|  | III-IV | 20 (17.5 %) | 10 (8.8 %) | 1 (0.9 %) |
| MMR status | MMR deficient | 47 (41.2 %) | 21 (18.4 %) | 2 (1.8 %) |
|  | MMR proficient | 30 (26.3 %) | 10 (8.8 %) | 3 (2.6 %) |
| p53 status | p53 aberrant | 32 (28.1 %) | 15 (13.2 %) | 2 (1.8 %) |
|  | p53 wild type | 45 (39.5 %) | 15 (13.2 %) | 3 (2.6 %) |
| Ki -67 status | Ki-67 ≥ 25 % | 18 (15.8 %) | 6 (5.3 %) | 1 (0.9 %) |
|  | Ki-67 < 25 % | 52 (45.6 %) | 2 (1.8 %) | 4 (3.5 %) |
| Grading | G1 | 36 (31.6 %) | 12 (10.5 %) | 2 (1.8 %) |
|  | G2 or G3 | 41 (36 %) | 18 (15.8 %) | 3 (2.6 %) |
| Histopathology | Endometrioid carcinoma | 65 (57 %) | 26 (22.8 %) | 5 (4.4 %) |
|  | Others (e.g. serous or clear cell carcinomas) | 12 (10.5 %) | 5 (4.4 %) | 0 |

*statistically significant, *p* ≤ 0.05


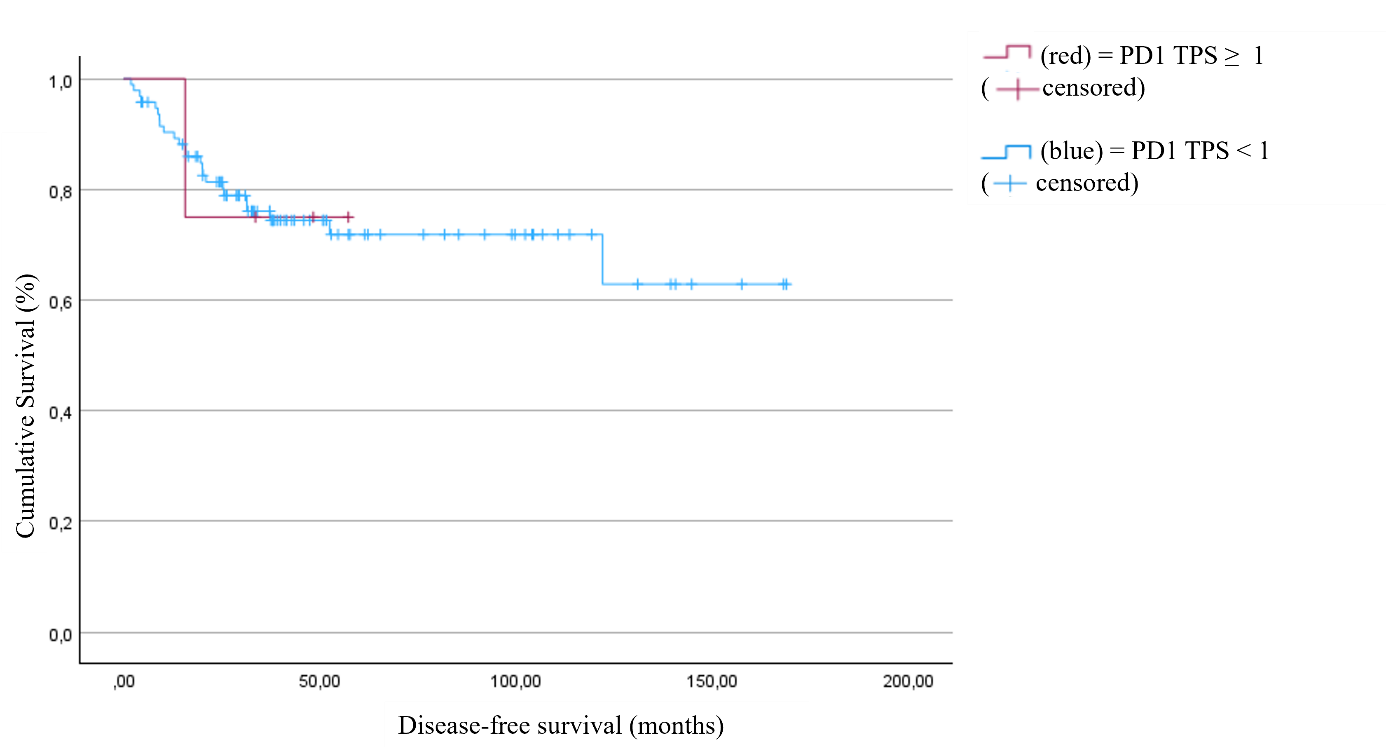


Fig. S1. Disease-free survival as a function of PD1 positivity, defined as TPS ≥ 1 (*p* = 0.983).


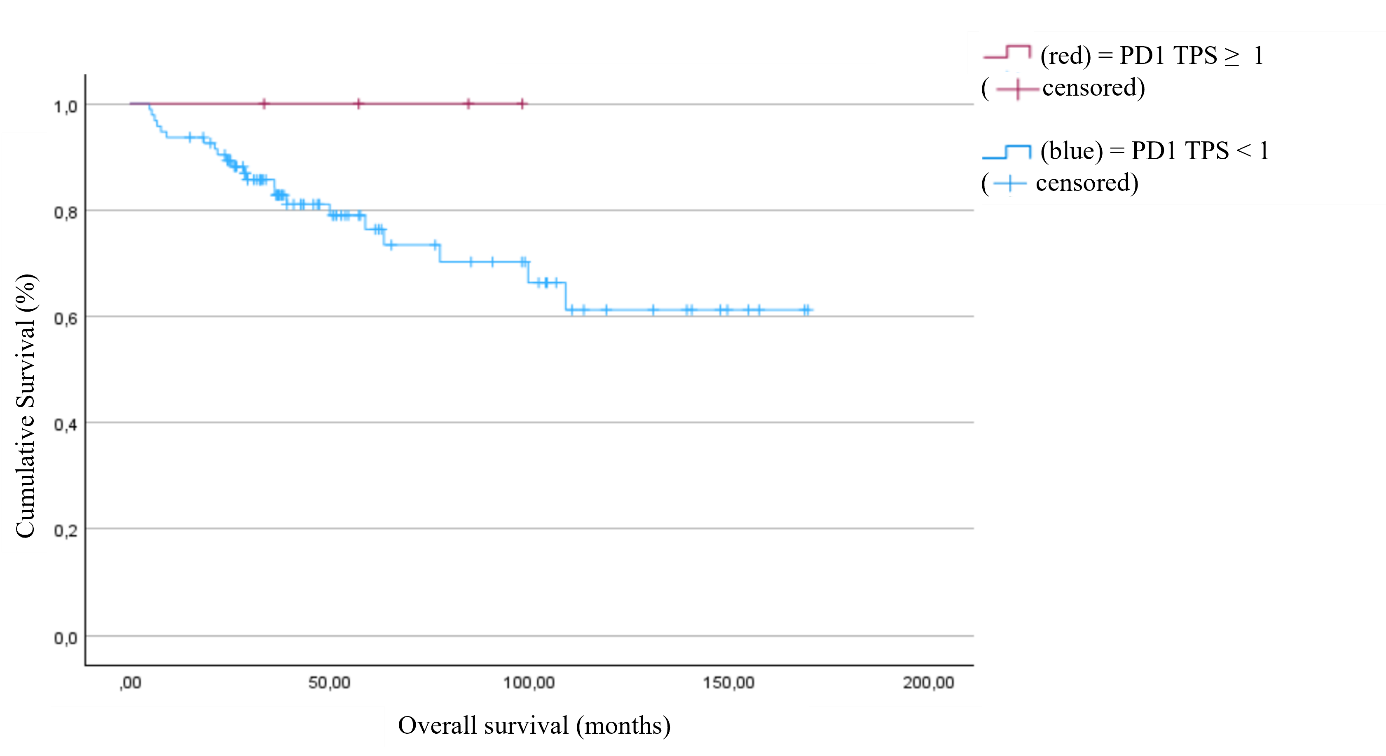


Fig. S2. Overall survival as a function of PD1 positivity, defined as TPS ≥ 1 (*p* = 0.289).


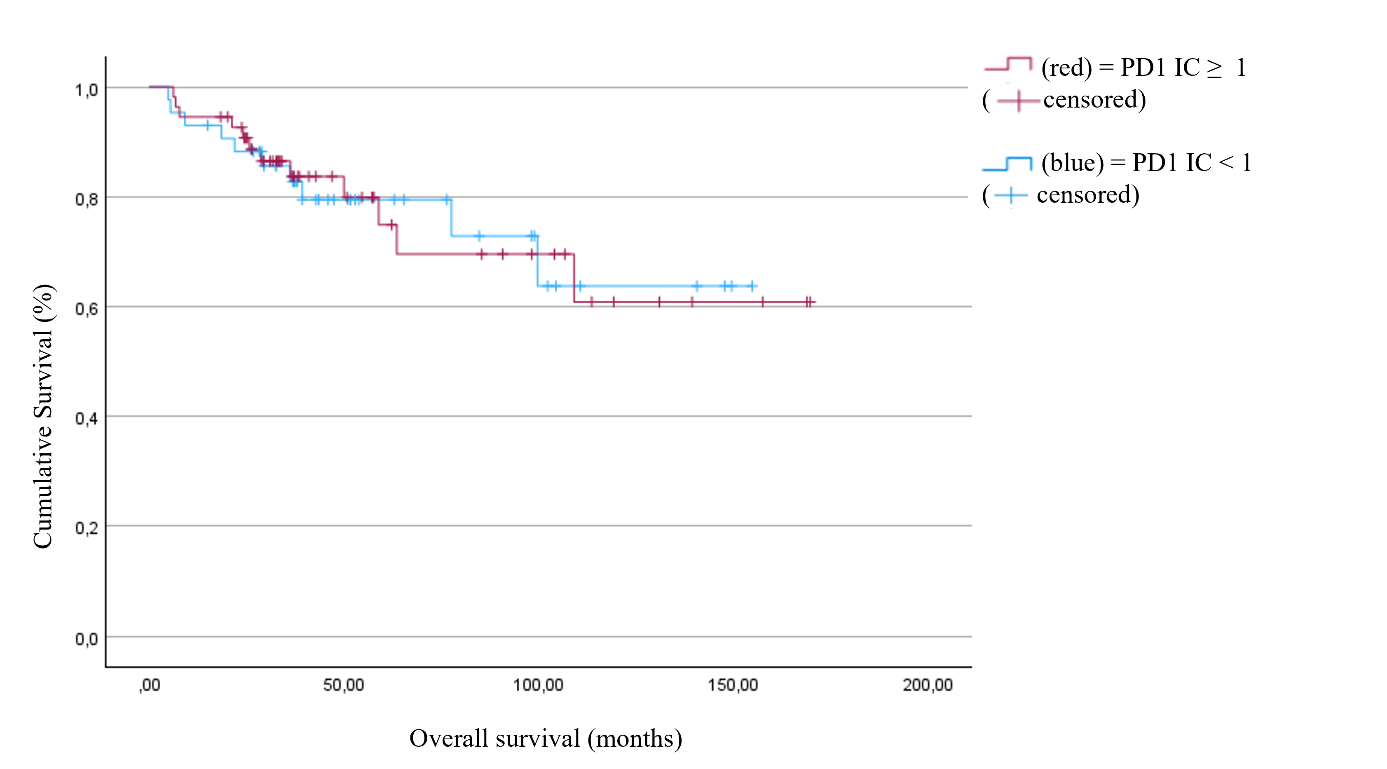


Fig. S3. Overall survival as a function of PD1 positivity, defined as IC ≥ 1 (*p* = 0.986).


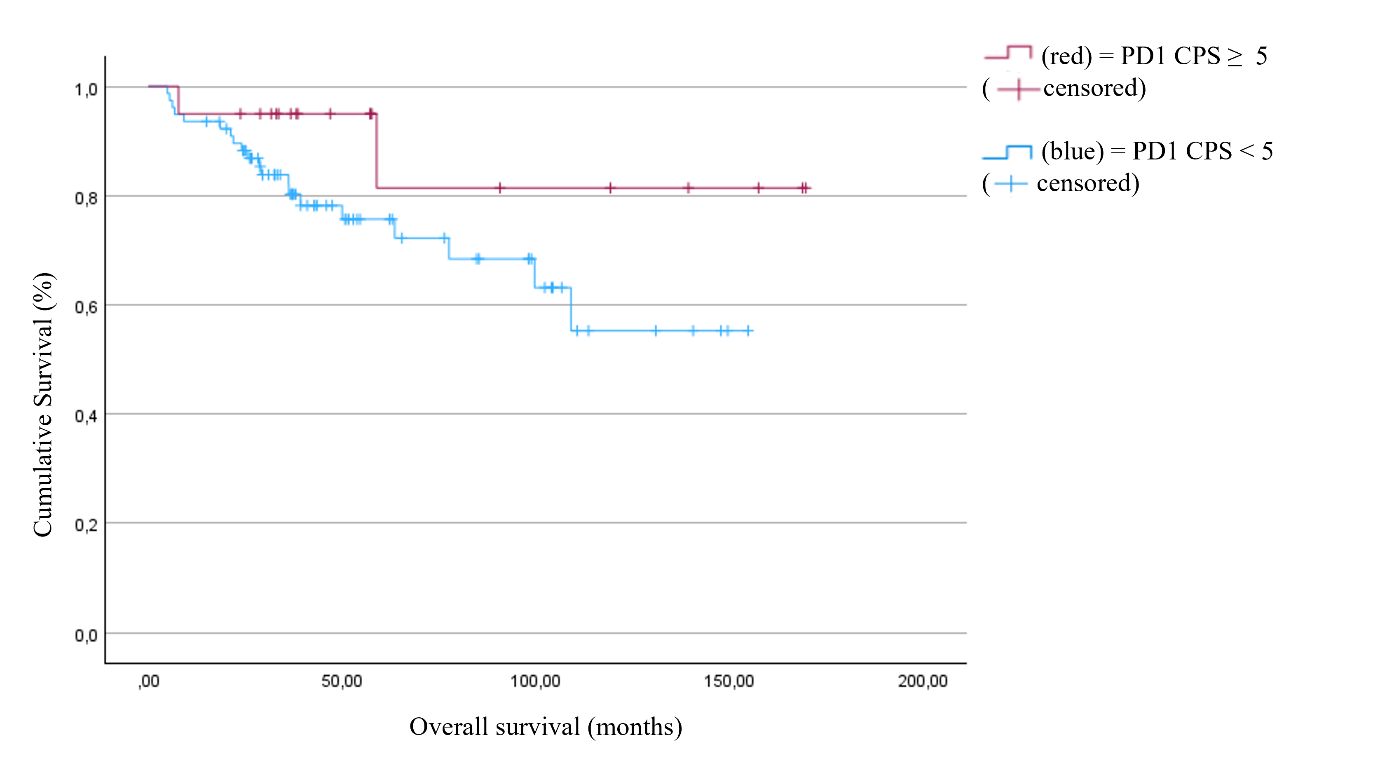


Fig. S4. Overall survival as a function of PD1 positivity, defined as CPS ≥ 5 (*p* = 0.119).

PD-L1-Positivity

The survival data related to PDL1-positivity are also provided by this supplemental information. These data do not yield significantly different results across the various PD-L1-scores. The Kaplan-Meier curves are illustrated below.


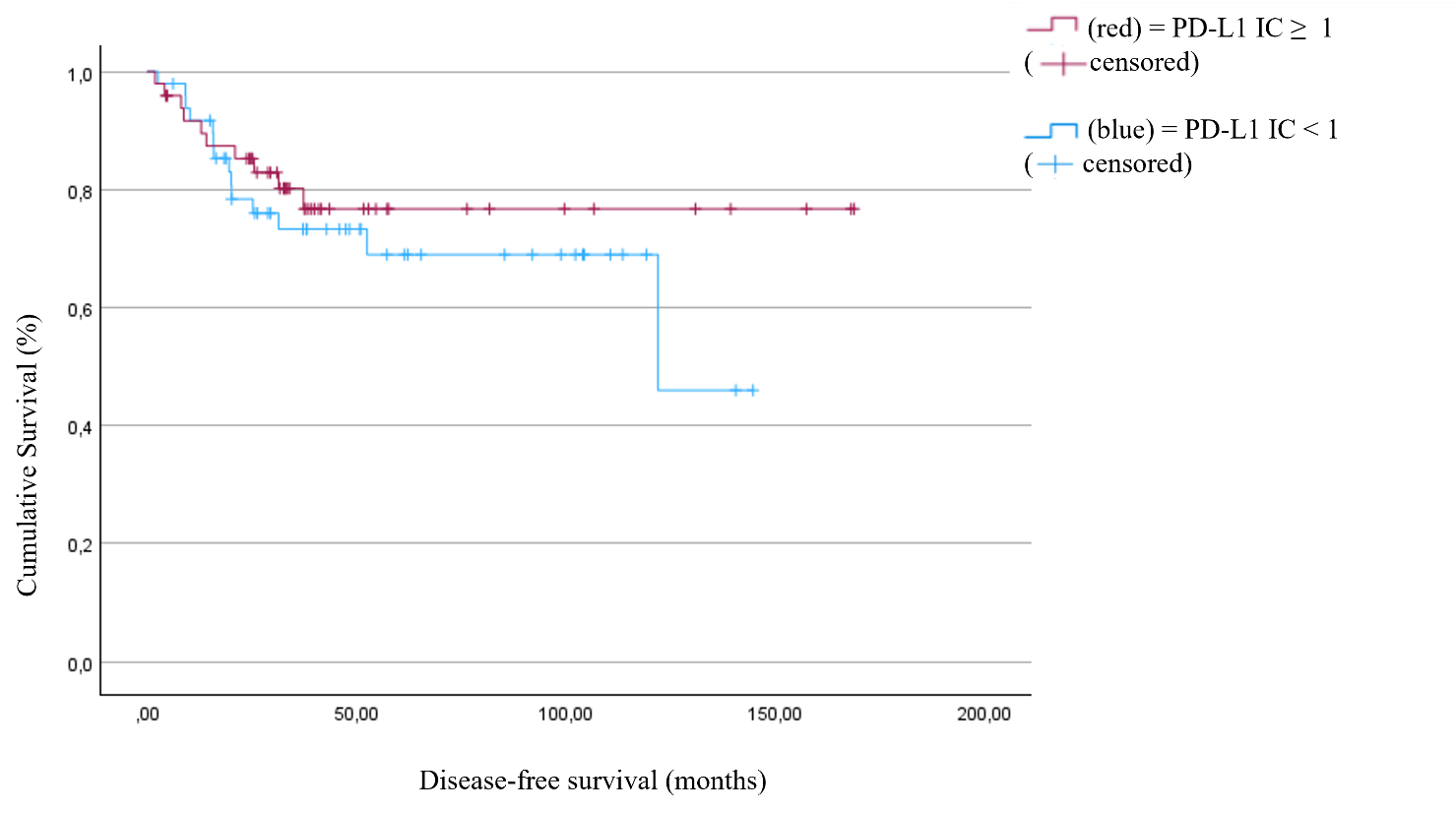


Fig. S5. Disease-free survival as a function of PD-L1 positivity, defined as IC ≥ 1 (*p* = 0.373).


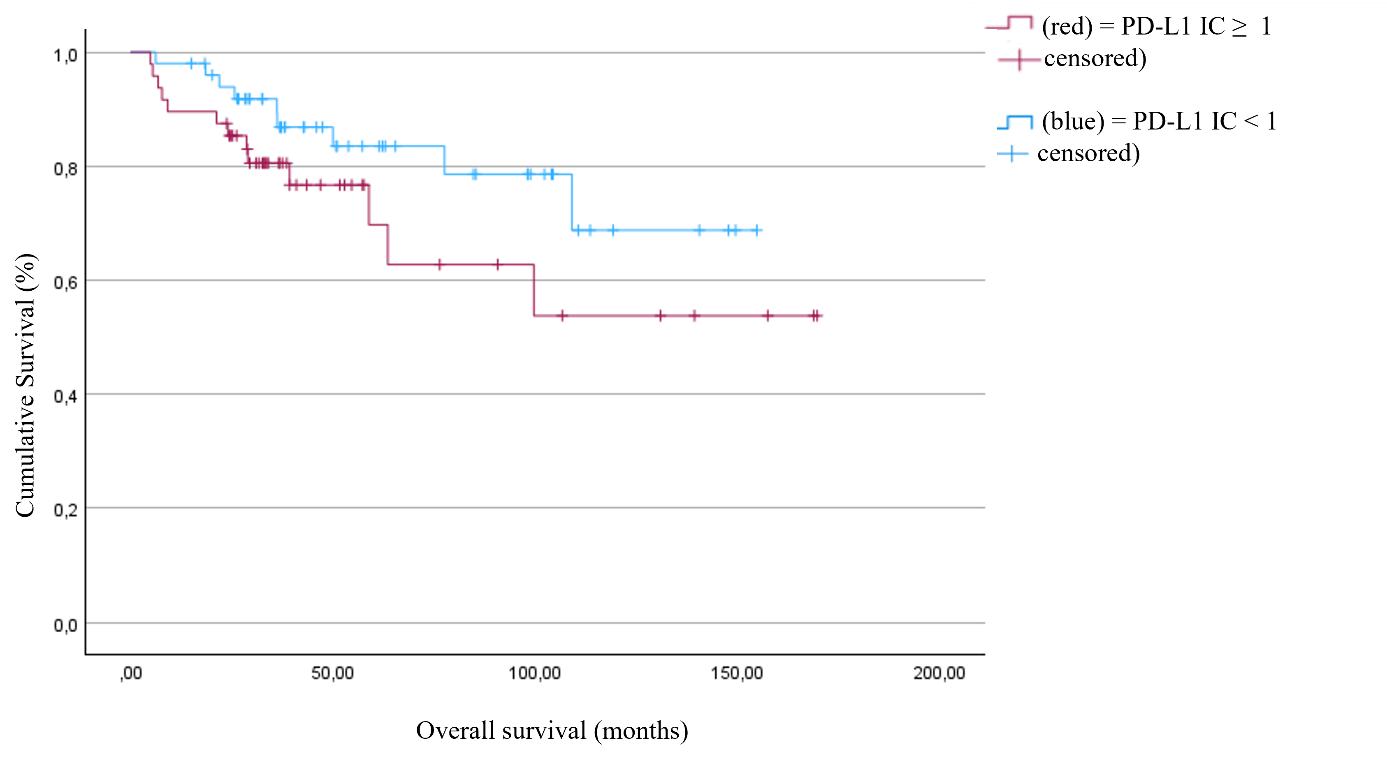


Fig. S6. Overall survival as a function of PD-L1 positivity, defined as IC ≥ 1 (*p* = 0.135).


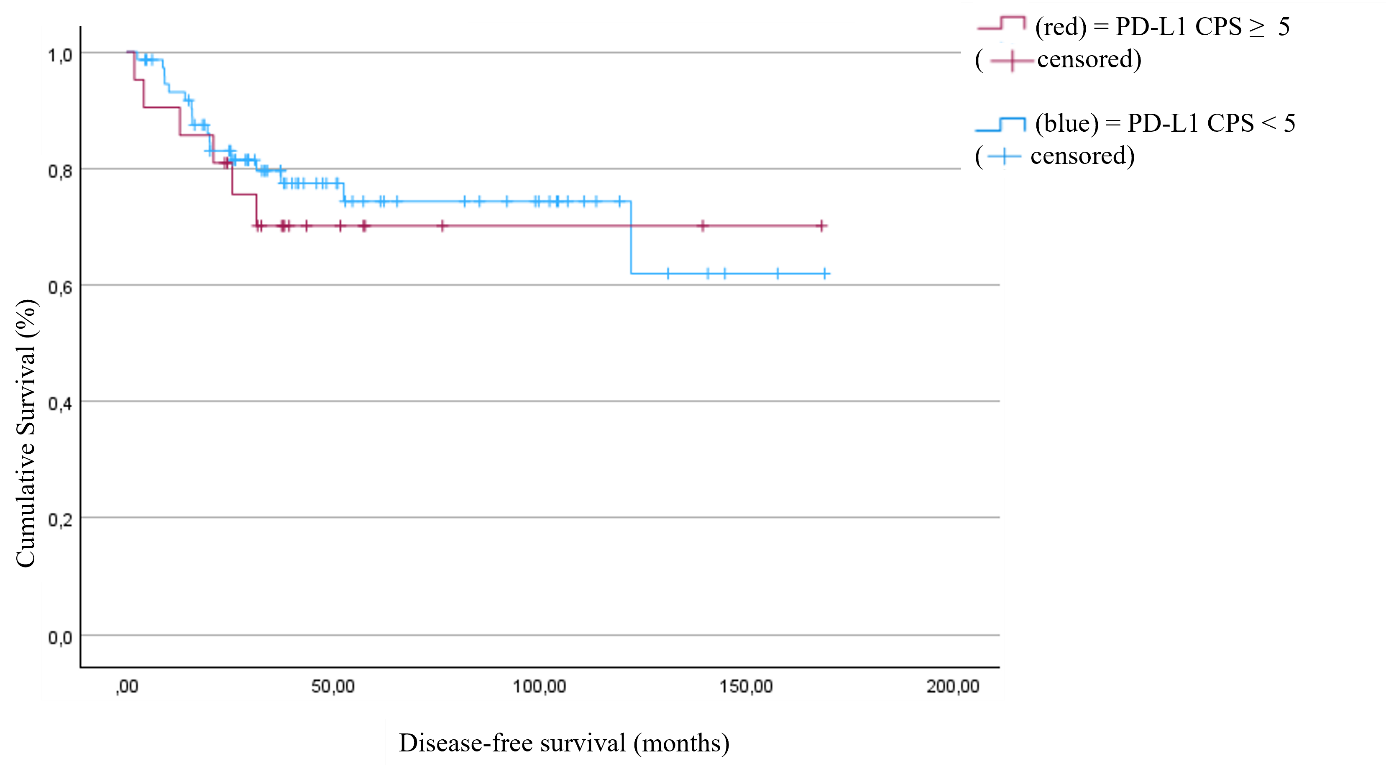


Fig. S7. Disease-free survival as a function of PD-L1 positivity, defined as CPS ≥ 5 (*p* = 0.660).


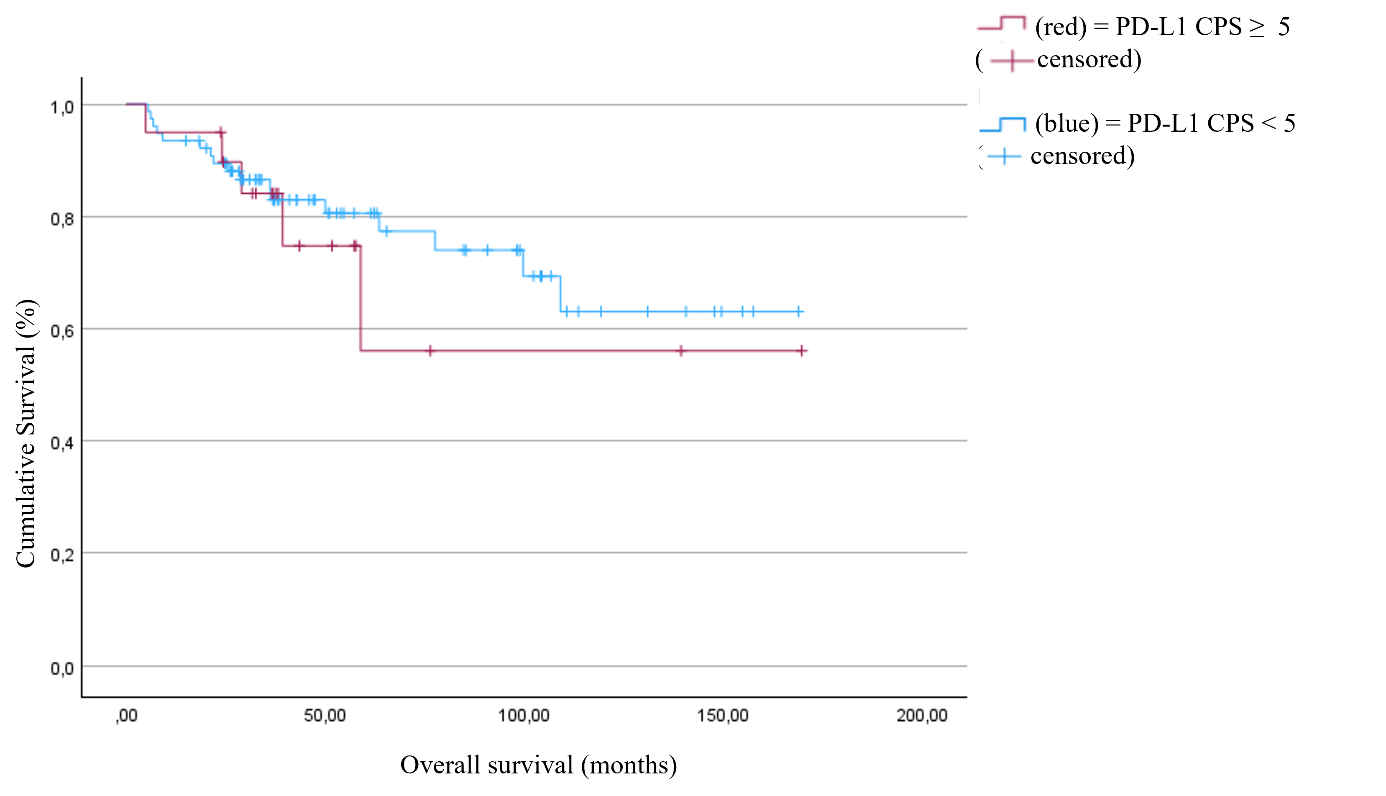


Fig. S8. Overall survival as a function of PD-L1 positivity, defined as CPS ≥ 5 (*p* = 0.609).


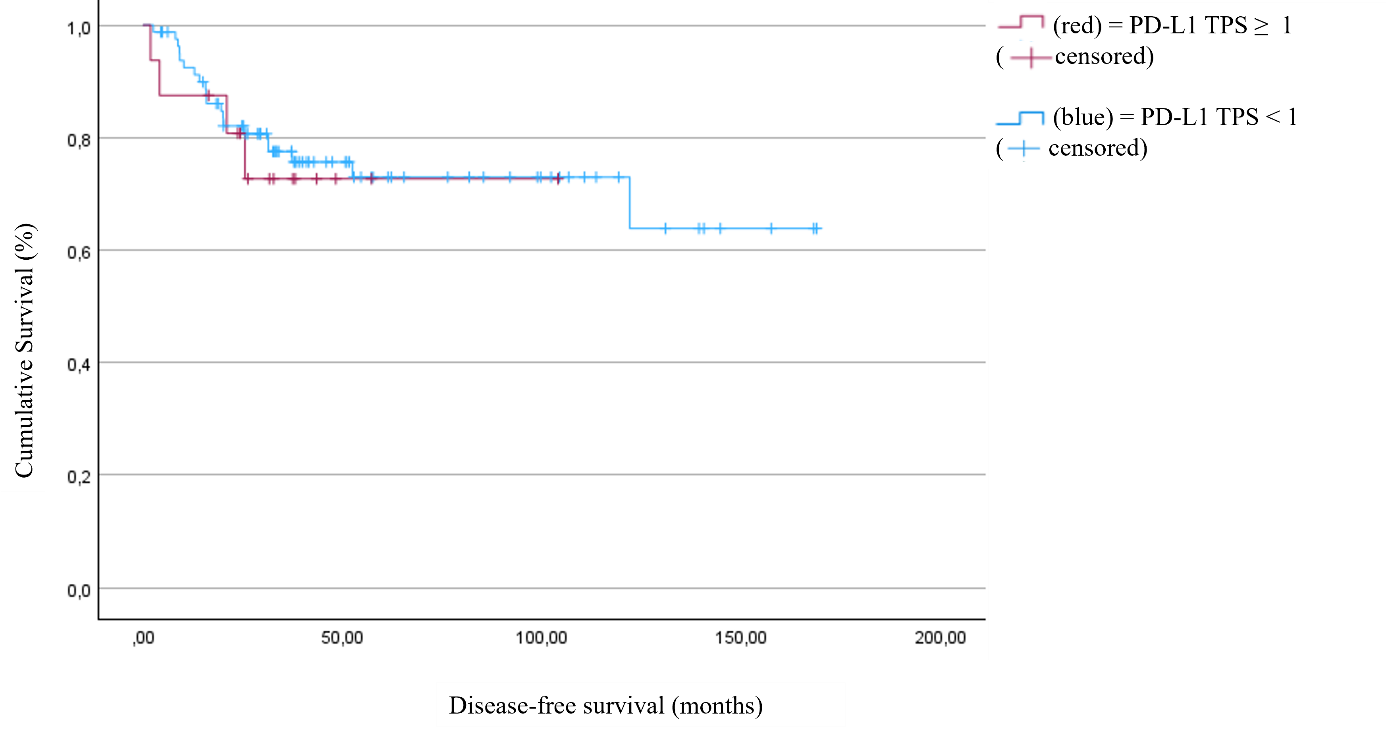


Fig. S9. Disease-free survival as a function of PD-L1 positivity, defined as TPS ≥ 1 (*p* = 0.780).


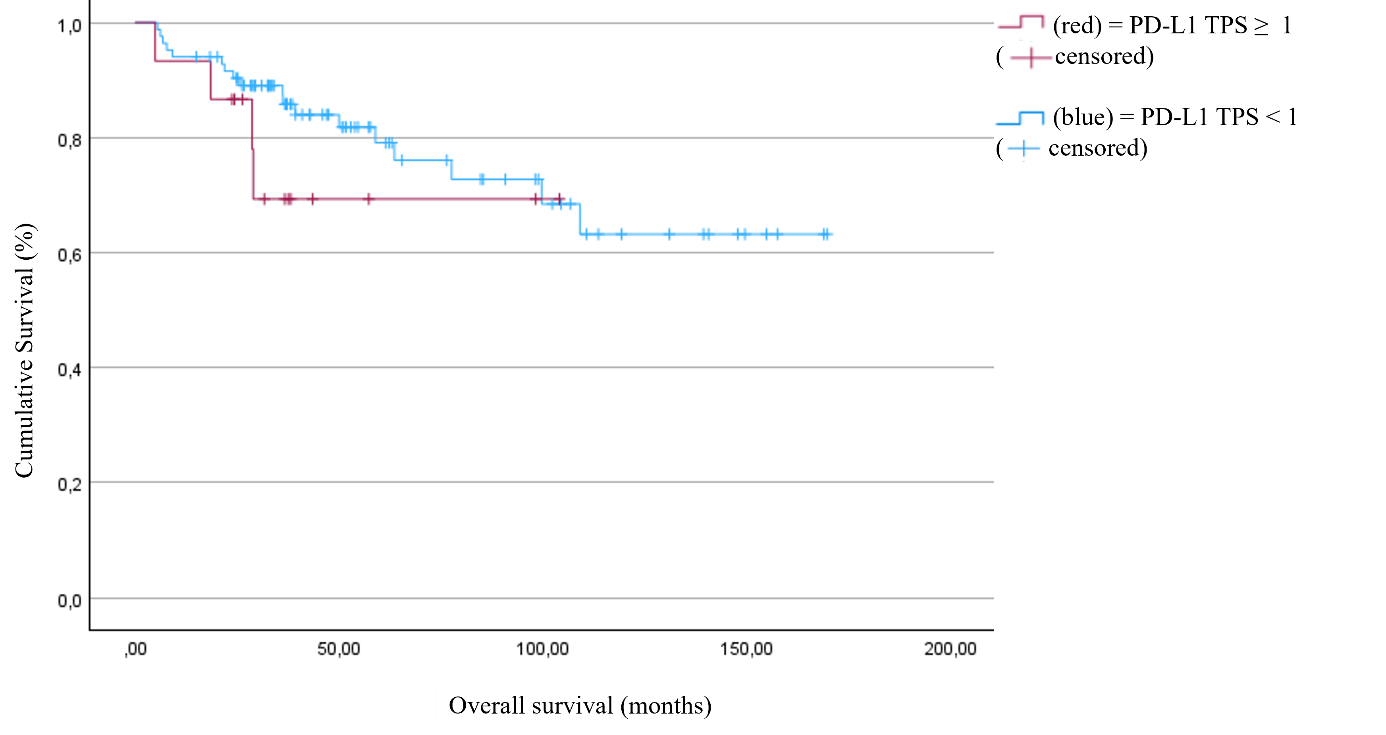


Fig. S10. Overall survival as a function of PD-L1 positivity, defined as TPS ≥ 1 (*p* = 0.310).
